# Identification of a novel mutation in CYBB gene in a Chinese neonate with X-linked chronic granulomatous disease: A case report

**DOI:** 10.1097/MD.0000000000028875

**Published:** 2022-03-11

**Authors:** Jie Zhang, Meili Fan, Mengmeng Chen, Huihui Wang, Na Miao, Haihua Yu, Lehai Zhang, Qianqian Deng, Changying Yi

**Affiliations:** a Organization and Personnel Section, Jinan Children's Hospital (Children's Hospital of Shandong University), Jinan 250022, China; b Enuresis Clinic of Tuina Department, Jinan Children's Hospital (Children's Hospital of Shandong University), Jinan 250022, China; c Clinical Laboratory, Jinan Children's Hospital (Children's Hospital of Shandong University), Jinan 250022, China; d Trauma Center, Shandong Provincial Hospital affiliated to Shandong First Medical University, Jinan 250021, China; e Department of Obstetrics, Shandong Provincial Hospital Affiliated to Shandong First Medical University, Jinan 250021, China; f Neonatal Intensive Care Unit, Jinan Children's Hospital (Children's Hospital of Shandong University), Jinan 250022, China; g Obstetrics and Gynecology Department, Lingcheng District's Traditional Chinese Medicine Hospital, Dezhou 253500, China.

**Keywords:** chronic granulomatous disease, CYBB gene, mutation, next-generation sequencing

## Abstract

**Rationale:**

X-linked chronic granulomatous disease (X-CGD) is an X-linked recessive disorder of the Nicotinamide adenine dinucleotide phosphate oxidase system that can cause primary immunodeficiency. Mutations in the *CYBB* gene located in Xp21.1 were accounting for X-CGD disease. More than 600 mutations have been identified as the cause of X-CGD in various populations worldwide.

**Patient concerns and diagnosis:**

In this study, the proband suffered from elevated white blood cells (WBC, 23.65 × 109/L), mainly in neutral (16.4 × 109/L). The neutrophil oxidative index of the patient was 2.13, which was extremely low, whereas his mother was 69.0 (Ref >100). Next, next-generation sequencing of the primary immunodeficiency diseases -related gene panel was performed. One novel mutation was identified in the CYBB gene in the CGD case: c.55C>G in exon 2. The mutation was verified by Sanger sequencing. The mother of the patient was heterozygous for the c.55C>G mutation, and the father was normal. These mutations were not present in the 100 unrelated normal controls.

**Interventions and outcomes:**

The patient died from severe and uncontrollable pulmonary infection at 3 months of age.

**Lessons:**

The identification of these mutations in this study further expands the spectrum of known CYBB gene mutations and contributes to the genetic counseling and prenatal molecular diagnosis of X-CGD.

## 1 Introduction

Chronic granulomatous disease (CGD) is a rare inherited disorder with a prevalence of 1/200,000–250,000 and is characterized by early and recurrent severe bacterial or fungal infections and granuloma formation,^[1]^ and the prevalence rates of CGD are similar in different ethnic and racial crowds.^[2]^ CGD is a primary immunodeficiency disorder caused by damage to the Nicotinamide adenine dinucleotide phosphate oxidase system. The Nicotinamide adenine dinucleotide phosphate oxidase system consists of 5 subunits: gp91phox (also called NOX2, encoding gene CYBB), p22phox (encoding gene CYBA), p47phox (encoding gene NCF1), p67phox (encoding gene NCF2), and p40phox (encoding gene NCF4). Mutations in CYBB account for two-thirds of CGD cases.^[2,3]^ The CYBB gene located in Xp21.1 could cause X-linked CGD (X-CGD, MIM: 300481) disease.

To date, at least 648 CYBB mutations have been reported worldwide, and approximately one-third of CYBB mutations occur de novo (Human Gene Mutation Database. http://www.hgmd.cf.ac.uk/ac/index.php).^[4]^ In the Chinese population, one previous study reported 38 children with chronic granulomatous disease in Chongqing Children's Hospital, and 19 novel mutations of the CYBB gene were discovered,^[5]^ while Beijing Children's Hospital collected 12 boys with X-CGD, and 8 novel mutations were found.^[6]^ However, there are fewer papers on CGD mutations in China than in other countries.

In the present study, we used a sensitive and specific Next Generation Sequenci600PEI cell (NGS) method for the identification of mutations in primary immunodeficiency disease (PID)-related genes from Mygenostics and found a novel CYBB mutation in a child from Shandong Province, North China.

## 2 Methods

### 2.1 Gene mutation analysis

All experiments involving blood collection from the family and 100 normal controls were approved by Jinan's Children's Hospital Medical Ethics Committee. Informed consent was obtained from all the participants for participation and publication of the study.

Peripheral blood samples were collected from the family and healthy controls for DNA extraction using a DNA extraction kit (TIANamp Blood Genomic DNA Purification Kit; Tiangen Biotech, Beijing, China). Genomic DNA products were purified according to the manufacturer's instructions (concentration= 100–150 ng/μL; OD260/OD280 = 1.7–1.9).

High-throughput DNA sequencing and NGS were used for mutation screening of the proband. A total of 239 genes associated with PID, including protein-coding regions, were selected using a gene capture strategy with a GeneCap custom exome enrichment kit (Mygenostics, Beijing, China). 15 ug of DNA from the proband were used to generate index libraries (average size 350–450 bp, including adapter sequences) for Solexa hiseq2000 sequencing (Illumina, San Diego, CA). The sequencing was performed for 90 cycles per read. The obtained mean exome coverage was >98%, with an accuracy of >99%.^[7,8]^ Sanger sequencing was used to validate the identified potential disease-causing variants in the family, including his parents and 100 unrelated normal controls.

### 2.2 Bioinformatic analysis of mutation

Homologous protein sequence alignments and bioinformatics analysis of the c.55C>G mutation were conducted. Potential effects of the mutation on function were assessed using the following software: UCSC Genome Bioinformatics (http://genome.ucsc.edu/), Human Gene Mutation Database (http://www.hgmd.cf.ac.uk/ac/index.php), PolyPhen-2 database (http://genetics.bwh.harvard.edu/pph2/index.shtml), and ClustalX software.

### 2.3 Case presentation

One 1-month-old boy presented with recurrent and persistent pneumonia, and thorough examination revealed that he was highly suspected to have CGD. His parents were non-consanguinous and the family belonged to Han Chinese ethnicity.

### 2.4 Patient's characteristics

The patient was admitted to the hospital with persistent fever and pneumonia. Clinical examination revealed that the child had normal mental status, no cough and expectoration, rough breath phonic of double lung without pleural friction sound, normal heart rate and bowel sounds, and normal liver and spleen. Chest radiography and computed tomography showed pneumonia, multiple atelectasis in the bilateral lung, pulmonary consolidation, and local liquefaction necrosis. Endoscopic examination showed no obvious mucosal erosion or embolization and no caseous necrosis or growth of granulation tissue. *Staphylococcus haemolyticus* was isolated from blood cultures.

The laboratory results showed that serum IgG (24.1 g/L), IgM (1.62 g/L) were increased, and the proband suffered from elevated white blood cell (23.65 × 109/L), mainly neutrophils (16.4 × 109/L). The neutrophil oxidative index of the patient was 2.13, that of his mother was 69.0, and that of his father was normal. The score in the normal control group was >100. The patient died from a severe and uncontrollable pulmonary infection at 3 months of age.

### 2.5 Mutation analysis

**Figure 1:**
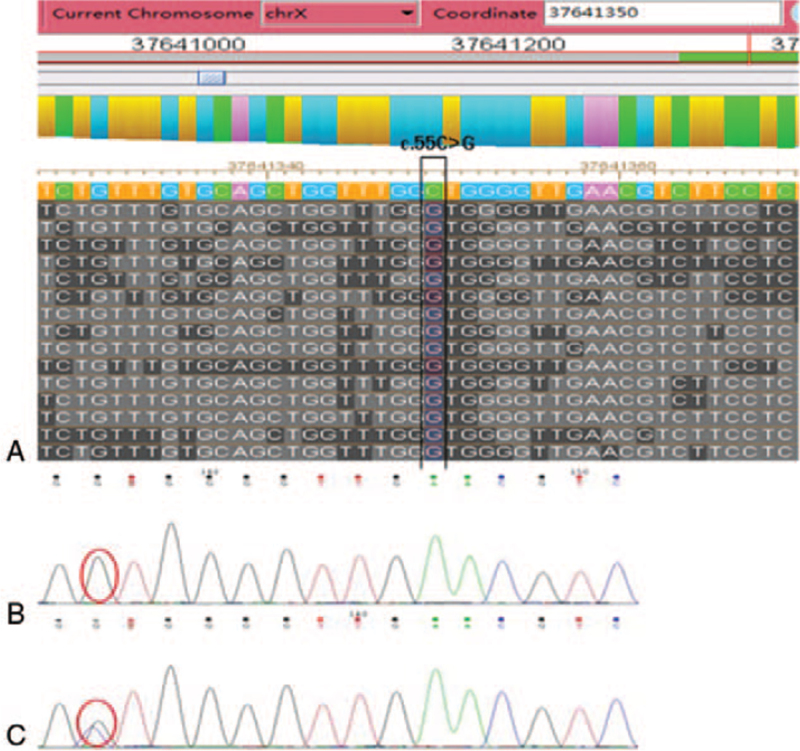
CYBB gene mutation in the proband compared with his parents. A, CYBB gene sequence in the proband by NGS. B, CYBB gene sequence in the proband by sanger sequencing. C, CYBB gene sequence in the mother by sanger sequencing.

Detection of mutations in PID-related genes through NGS in the proband revealed one novel mutation: c.55C>G (p.Leu19Val; ab. p.L19V) in exon2 (Fig. 1). The mutation was verified by Sanger sequencing, and the mother of the patient was heterozygous for the c.55C>G mutation, while the father was normal (Fig. 1).

### 2.6 Bioinformatics analysis

**Figure 2:**
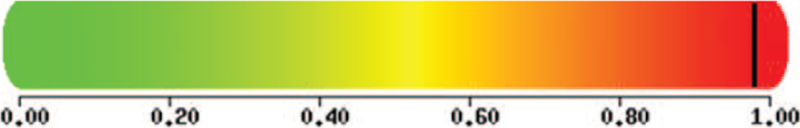
PolyPhen-2 report for P04839 L19V mutation. This mutation is predicted to be PROBABLY DAMAGING with a score of 0.978 (sensitivity: 0.76; specificity: 0.96).

**Figure 3:**
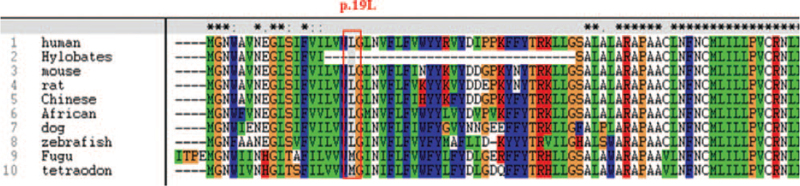
The p.L19 site of orthologous CGD amino acid sequences from 10 species was found to be highly conserved.

To further characterize the c.55C>G mutation, we conducted bioinformatics analysis (Fig. 2). The p.L19 site was highly conserved in orthologous NOX2 proteins of multiple species (Fig. 3). The functional sites of the CYBB protein were analyzed using the PolyPhen-2 database. The results showed that the CYBB protein contained 6 transmembrane domains, and p.L19 was located in the first transmembrane domain including 9-29 amino acids. P.L19 possibly played a role in the formation of transmembrane helices, so the mutation p.L19V possibly damaged protein function.

## 3 Discussion

Congenital phagocyte deficiency diseases are a part of primary immunodeficiency disease (PID),^[9]^ in which X-CGD is the most common disease. To date, at least 648 CYBB mutations have been reported (in HGMD database). Gene mutations with significant heterogeneity were widely distributed in 13 exons. The novel mutation c.55C>G was first identified and it enriched the CYBB gene mutation database. The protein NOX2 encoded by the CYBB gene contains 6 hydrophobic transmembrane domains, containing 2 heme groups within the N-terminal domain, and 338–344 amino acids have nucleotide-binding positions.^[10,11]^ p.L19 is located in the first transmembrane domain. Leucine residues, including p.L19, are important for maintaining the normal structure and function of the protein. This change in p.L19V may have damaged the helical framework. The p 19 L position of orthologous NOX2 proteins in different species was well conserved, except for inferior creatures with p.19M.

Patients with X-CGD usually have an early onset of atypical clinical manifestations and hidden infection. Despite complicated multisite infections, the main infection was pneumonia. Accurate diagnosis of the disease is difficult. The laboratory results showed hypergammaglobulinemia and elevated leukocytes, which gave a hint of CGD. CGD can be screened using NBT testing. Moreover, the neutrophil respiratory burst test is a quick and effective method for diagnosing CGD. Neither of them could differentiate CGD. Genetic diagnosis has provided valuable methods for identifying both patients and carriers.^[4,12,13]^

Next-generation sequencing (NGS) methods^[14]^ have become increasingly accessible for clinical laboratory diagnoses. These methods have the advantages of being rapid, accurate, and relatively inexpensive. While NGS is not as sensitive as Sanger sequencing for individual genes, targeted NGS has become a cost-effective first-line genetic test for the evaluation of patients with single-gene disorders, especially PIDs.^[9]^ In our research, NGS increased the diagnostic rate and provided insight into the genotype–phenotype correlation of genetic diseases in a cost-effective way.

At present, there are no specific treatments for CGD, and many patients die of severe infections.^[15]^ Our patient died from a severe pulmonary infection at 3 months of age. All the aforementioned results indicated that the c.C55G mutation was likely a pathogenic mutation. Gene mutation analysis of CGD is an important tool for genetic counseling and prenatal diagnosis.^[16]^

## Acknowledgments

The authors gratefully acknowledge the participation and cooperation of patients with X-CGD in this study.

## Author contributions

**Data curation:** Huihui Wang, Na Miao.

**Formal analysis:** Jie Zhang, Lehai Zhang.

**Investigation:** Haihua Yu, Mengmeng Chen

**Methodology:** Jie Zhang, Changying Yi, Qianqian Deng.

**Supervision:** Changying Yi, Meili Fan.

**Writing – review & editing:** Changying Yi, Meili Fan.
